# Demographic and genetic structure of a severely fragmented population of the endangered hog deer (*Axis porcinus*) in the Indo-Burma biodiversity hotspot

**DOI:** 10.1371/journal.pone.0210382

**Published:** 2020-02-06

**Authors:** Sangeeta Angom, Chongpi Tuboi, Mirza Ghazanfar Ullah Ghazi, Ruchi Badola, Syed Ainul Hussain

**Affiliations:** Wildlife Institute of India, Dehra Dun, Uttarakhand, India; National Cheng Kung University, TAIWAN

## Abstract

The population of the globally endangered hog deer (*Axis porcinus*) has declined severely across its geographic range. Intensive monitoring of its demographic and genetic status is necessary. We examined the demographic and genetic structure of a small hog deer population in Keibul Lamjao National Park (KLNP), located on the western fringe of the Indo-Burma biodiversity hotspot for conservation planning. The distribution pattern of hog deer in the Park was derived based on the presence/absence of faecal pellets in 1 km × 1 km grids. We used double-observer distance sampling method to derive the hog deer abundance and population structure and compared with previous data to derive the population trend. We determined the genetic diversity of the population through microsatellite screening and bottleneck detection. The overall pellet density was 0.34 ± 0.02 pellets km^-2^ restricted to only 22.34 ± 0.20 km^2^ area of the park. The estimated density of the deer in the park was 1.82–4.32 individuals km^-2^. The population showed a declining trend from 2006–08 (*p* < 0.05, R^2^ = 0.916) with 8% annum^-^^1^ and an increasing trend from 2003–2018 (*p* < 0.05, R^2^ = 0.9304) with 10% annum^-^^1^. The adult male-to-female ratio and fawn-to-doe ratio were 36.2 ± 1.9 males per 100 females and 16.5 ± 0.4 fawns per 100 females, respectively. The molecular examination suggested that the mean number of alleles at 23 loci was 2.70 ± 0.18, the observed heterozygosity (Ho) ranged from 0.26 to 0.63 (mean 0.42 ± 0.02), the expected heterozygosity (He) ranged from 0.23 to 0.73 (χ = 0.51 ± 0.03), and the polymorphic information content (PIC) ranged from 0.2 to 0.67 (χ = 0.43 ± 0.03) indicating a moderate level of genetic diversity. Although no bottleneck in the population was observed, the loss of genetic diversity may affect the evolutionary potential of the species at the site by limiting the selection flexibility. Conservation planning coupled with scientific management regime will help in the long term persistence of the population in the region.

## Introduction

The hog deer (*Axis porcinus*) is an ‘endemic’ species, geographically confined to South and Southeast Asia. There are two subspecies—the Southeast Asian subspecies (*A*. *p*. *annamiticus*) and the Indian subspecies (*A*. *p*. *porcinus*). The Southeast Asian subspecies originally occurred in Thailand, China, Laos PDR, Vietnam and Cambodia whereas the Indian subspecies occurred in India, Nepal, Bangladesh, Bangladesh and Burma [1]. It was once widespread, but the population has declined rapidly across its geographic range. It is estimated that the global decline rate over the last 21 years is 50% and that the species has declined by more than 90% within its Southeast Asian range [2]. The species has completely disappeared from Thailand, Laos and Vietnam [2].

In India, the species is distributed throughout the northern plains and in the Northeast region [3]. The latter has emerged as a stronghold for the species, with large local subpopulations in Assam and West Bengal [4] and in Manipur, where a small, secure subpopulation survives [5, 6]. Nevertheless, the species is estimated to have undergone a decline of 30–40% or more in the country over the last 21 years [7].

On the basis of these estimates (the average for the species in three generations), the species has been listed as “Endangered” in the IUCN Red List [7]. Hunting, habitat loss and habitat degradation have been the major drivers of the decline [2]. The extant population is now patchily distributed and highly fragmented [8]. The relict populations are found in low alluvial grasslands in the Brahmaputra flood plains and in Nepal, Bangladesh and Southwest Yunnan Province, in China [2], and are probably suffering from inbreeding depression [9, 10].

Phylogenetic analysis using both the mitochondrial and the nuclear DNA suggests that the hog deer belongs to the subfamily Cervinae [11]. Little empirical information is available on the ecology and conservation genetics of this species. In order to design successful and effective conservation strategies for this species, information about its genetic variability and structure is crucial.

Northeast India is particularly significant in terms of the lineage and genetic diversity of the hog deer, being a stronghold of the species [4] and possibly holding a link between the Indian subspecies and the Southeast Asian subspecies. The localised and isolated hog deer populations in the state of Manipur are not well studied, and, therefore, little is known about the status of these populations and their genetic structure. Recent studies have also revealed that the hog deer population in Keibul Lamjao National Park, Manipur is the Southeast Asian subspecies (*A*. *p*. *annamiticus*) [12]. Isolated populations such as these are likely to be more vulnerable to local extinction because they are at higher risk of genetic, demographic and environmental stochasticity [10, 13, 14, 15]. Understanding the population dynamics of any species requires a combination of demographic, genetic and ecological studies. The effective population size and the number of individuals that actually contribute to the gene pool are key factors in the dynamics [16]. In view of the aforementioned, this research was carried out to examine the demographic status and genetic structure of the hog deer in Keibul Lamjao National Park (KLNP), Manipur, India.

## Materials and methods

### Study area

The study was conducted in KLNP, Manipur, India, located on the south-eastern fringes of Loktak Lake, which is also a Ramsar site [17]. KLNP extends between latitudes 24°27′N and 24°31′N and longitudes 93°53′E and 93°55′E, in the Barak-Chindwin river basin. Loktak Lake is a wetland of international importance (Ramsar site) [18]. Presently the park has an extent of 40.05 km^2^. The characteristic floating meadows, locally known as *phumdis*, covers 26.41 km^2^ and the remaining extent of 14.09 km^2^ consists of open water, dry lands and hills [17]. The floating meadows of the park are of varying thickness, and they support small populations of the hog deer and Eld’s deer (*Rucervus eldii*). The change in the hydrology of the park due to the construction of the Ithai barrage in the downstream of Manipur River, in the early 1980s, has resulted in a rapid change in the Lake ecosystem and the natural process of meadow formation [17]. The meadows, that used to settle down during the lean season on the Lake bed and get replenished with soil and nourishment, are now continuously floating due to permanent flooding of the Park. Consequently, the thickness of the *phumdis* is decreasing at a rapid rate, which is detrimental, as the thickness has to be sufficient to support the weight of the wild ungulates such as hog deer and Eld’s deer, which are found sympatrically in the park.

The park experiences three distinct seasons, summer, winter and monsoon [18]. The floating meadows supports around 185 species of herbs, grasses and sedges of which *Phragmites karka*, *Leersia hexandra*, *Capillipedium* spp., *Zizania latifolia* and *Hemarthria compressa* contribute to the main food plants of the ungulates in the park [6, 19].

### Field methods

The entire park was divided into 1 km × 1 km grids to examine the distribution pattern and habitat use of hog deer. In each grid, based on the *phumdi* thickness and the presence of open water, plots of 50 m x 2 m were laid on transects of length 500 m (n = 66 in 2006; n = 75 in 2007; n = 72 in 2008), were laid and hog deer faecal pellet groups within each transect were recorded. Habitat parameters were recorded in circular plots of radius 2 m along each transect at 50 m intervals. The habitat parameters collected include ground cover (vegetation, litter, water and *phumdi*), substratum character (water/*phumdi*/soil), *phumdi* thickness, water depth, total number of faecal pellet groups, signs of associated species, ocular visibility, distance from uplands/hills, distance to water for drinking/wallowing and disturbance factors (fishing, grazing and vegetable collection).

A 0.5 m × 0.5 m quadrat was laid within each plot to record the number of each plant species present. On the basis of the distribution of the faecal pellet groups, the different areas of the park were classified as high, medium and low-density areas. Boat surveys were carried out at different study sites in the park to identify specific locations that were suitable for constructing *machans* (temporary bamboo watch towers) for estimating and monitoring the hog deer population. This exercise provided baseline information for constructing *machans* and also helped classify the habitat into thick and thin *phumdis*. No *machan* was constructed in those blocks in which no faecal pellets were recorded. In total, twenty-two *machans* were constructed with an average height of 7 meters to facilitate the monitoring activity in the dense vegetation of the park.

The point count method [20, 21] was used to estimate the abundance of hog deer in a 20 km^2^ area of the park excluding the water bodies and thin *phumdis*. Eight *machans* were constructed in the high pellet density areas and seven *machans* in the medium- and low-pellet-density areas. The counting of animals was carried out in the month of March from the *machans* using 8 × 40 prismatic binoculars when the visibility in the park was in its maximum due to the phenological trait of the grasses in the area. It was done between 0530 and 0900 hours and between 1500 and 1730 hours for five consecutive days using a double-observer method developed to estimate detection probabilities and to increase the detection accuracy [22, 23]. During each point count, a designated “primary observer” indicates to the “secondary observer” all the animals detected, who then records all the detections. The observers take the primary and secondary roles alternately during the course of a survey. This approach permits estimation of observer-specific detection probabilities, and abundance estimation is carried out under the assumption that no animal goes undetected during the sampling periods. During the exercise, details such as number of individuals sighted, time of sighting, sighting distance, sighting angle, age, sex and habitat type were recorded. The animals were classified as adult male, sub-adult male, adult female, sub-adult female, juvenile and fawn [24, 25].

### Statistical analyses

The mean faecal pellet group density was calculated (number/km^2^) using MS Excel. The percentage areas of the faecal pellet densities (high, medium and low) were also calculated. The area was then stratified according to the percentage area and faecal pellet distribution map was generated using the ArcView GIS software (9.1). The habitat data were pooled into a single matrix. Continuous independent variables were first evaluated for independence using Pearson correlations. Multiple regression models were run using SPSS 16 [26] to find the best combination of habitat variables that predicted hog deer occurrence. The best combination was determined from the explained variance, overall *F* and regression diagnostics. The data were normalised using the Z-transform. The scaling of the habitat use can be represented by a linear model of the form Y = a + bx_1_ + cx_2_ + dx_3_…, where Y is the predicted density and x_n_ represents the habitat variables.

The Distance 6.0 software package was used to derive the abundance [27]. The hog deer density was calculated using maximum peak sightings for 30 minutes to avoid duplicate counts. The best-fitting model on the basis of the Akaike information criterion (AIC) [28] was used to derive the density. The exponential rate of increase was derived by regressing the natural log (ln) of the population density (hog deer km^-1^) against year. The exponential rate of increase (ṝ) is the slope of the regression line. The percentage change per year was calculated as percent change = (e^r^—1)*100 [29]. The population trend was calculated for the present study based on the data generated during this study (2006–08) and also from the data generated by the Manipur Forest Department for 2003–2018 “S1 Table”.

### Microsatellites genotyping

The total genomic DNA was extracted from tissue hair, antlers and faecal samples (n = 38) using Kit (QIAGEN), QIAamp® DNA Micro Kit (QIAGEN) and QIAGEN Stool Mini Kit, respectively, following the manufacturer’s protocol [30]. The DNA fragments were separated on an automated DNA sequencer (Applied Biosystems). Different individuals were genotyped as either homozygote or heterozygote on the basis of the band pattern shown for each microsatellite locus. GeneMapper v.3.7 software was used to analyze the allele frequencies, heterozygosity and other parameters of genetic variability. To reduce the occurrence of allelic dropout, a multi-tube approached was employed [31]. Two repeats were performed, and if there was a heterozygote in three of four repeats, the site was considered to be a heterozygous locus. The same procedure was followed for a homozygote loci [32].

The overall summary statistics were calculated for each locus: observed heterozygosity (Ho), expected heterozygosity (He), number of alleles per locus and effective number of alleles and polymorphic information content (PIC) were calculated using allelic frequencies in CERVUS 3.0 [33]. The software CERVUS 3.0 was also used to detect the presence of any matching genotype between sampled individuals [33, 34]. The P_ID_ and P_ID-Sibs_ were also calculated using the same software [35, 36]. GENEPOP 4.7 software was used to derive the deviations from the Hardy-Weinberg equilibrium (HWE) [37].

### Bottleneck detection

To test the evidence of recent population bottleneck, a comparison of the He of a sample with the heterozygosity that would be expected of a sample from a population at mutation drift equilibrium with the same size and allele number (Heq), was carried out to investigate the signatures of bottleneck events [38]. As the allele number decreases faster than the heterozygosity, a bottleneck is indicated by He being greater than Heq in subsequent generations [39]. Transient heterozygosity excess and mode‐shift tests were performed through 1000 simulations using the BOTTLENECK 1.2.02 software package [40], assuming that mutations of the microsatellite loci followed an infinite allele model (IAM), a stepwise mutation model (SMM) or a two-phase model (TPM). In TPM, it was assumed that 70% of the mutations consist of subsist of one step and 30% consist of multistep changes with a variance of 30. To determine if the heterozygosity excess was significant, the sign test, standardized differences test and Wilcoxon test were performed. The allelic distribution was determined to see if it was approximately L-shaped curve (as expected under a mutation-drift equilibrium) or not (recent bottlenecks provoke a mode shift) [39, 41].

## Results

### Distribution pattern of hog deer in the Park

Faecal pellets were recorded in 22.34 ± 0.20 km^2^ area in the park. The high-density area (9.31 ± 0.30 km^2^) had 15.9 ± 1.0 pellets km^-2^, the medium-density area (8.10 ± 0.40 km^2^) had 6.66 ±1.30 pellets km^-2^ and the low-density area (4.8 ± 0.6 km^2^) had 2.61 ±0.15 pellets km^-2^. The overall pellet density was 0.34 ± 0.02 pellets km^-2^. The hog deer habitat usage was best explained by the combination of *phumdi* thickness, vegetation cover and abundance of short grass (R^2^ = 0.423; F = 11.475, df = 50, *p* < 0.001). The estimated coefficient of habitat use showed that the preference for a specific habitat by hog deer increased with increasing *phumdi* thickness (β = 0.087 ± 0.05), decreasing vegetation cover (β = -0.142 ± 0.044) and increasing abundance of short grass (β = 0.124 ± 0.048). In other words, it showed that hog deer favoured areas with thick *phumdi*, less vegetation cover and dominated by short grasses.

### Population trend

**Fig 1:**
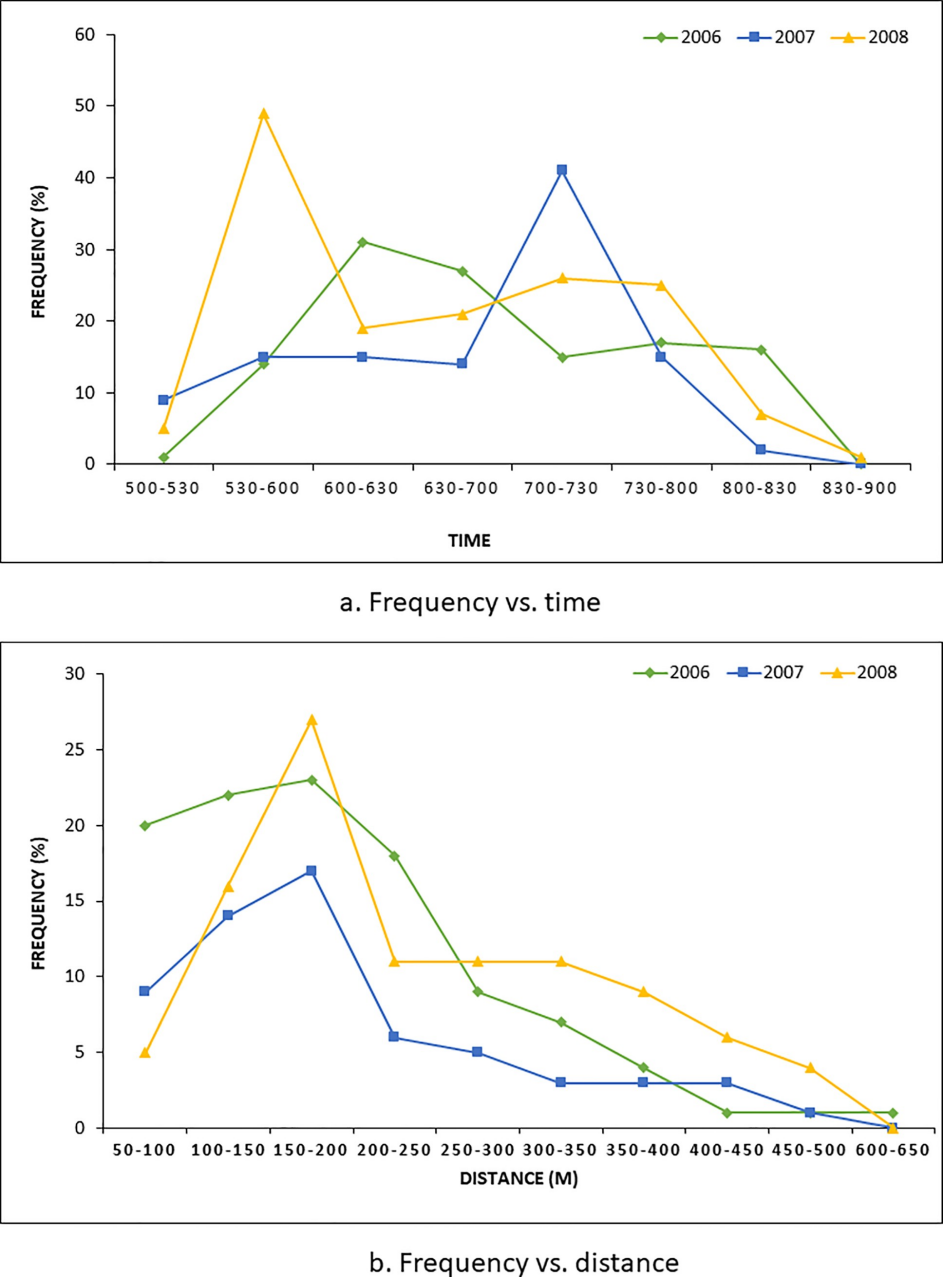
Frequency of sighting of hog deer during population estimation in Keibul Lamjao National Park, India. (a) frequency of sighting versus time and (b) frequency of sighting versus distance.

**Fig 2:**
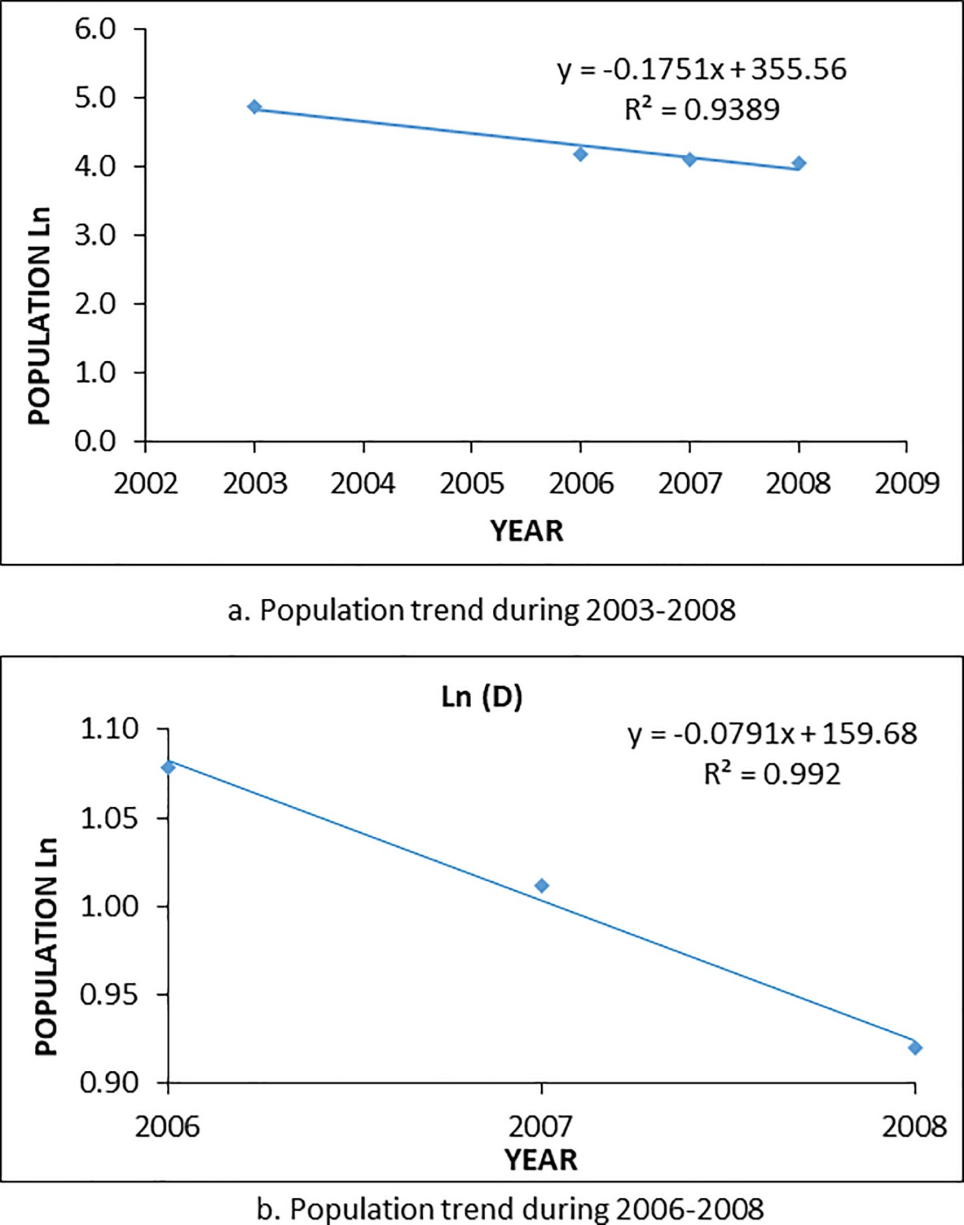
Population trend of hog deer in Keibul Lamjao National Park, India. (a) Population trend during 2003–2008 and (b) Population trend during 2006–2008.

Out of the eight models tested during the analysis using DISTANCE 6.0 software [42], based on the lowest AIC value, we used uniform cosine model to derive density of hog deer in the park “S2 Table”. The encounter rates were 0.36 ± 0.33, 0.22 ± 0.20 and 0.24 ± 0.20 individuals km^-2^ during 2006–2008 respectively and the probabilities of detection were 0.38 ± 0.58, 0.31 ± 0.29 and 0.30 ± 0.25, and the effective detection radius (EDR) values were 1.92 ± 0.35, 1.19 ± 0.17 and 1.25 ± 0.17, respectively, during the study period. The mean cluster sizes were 1.53 ± 0.10, 2.29 ± 0.18 and 2.02 ± 0.18 respectively, during the study period (Table 1). The estimated densities were 2.94 ± 0.57 (CV 19.5%), 2.75 ± 0.44 (CV 16.3%) and 2.51 ± 0.40 (CV 16.2%) individuals km^-2^ respectively, with a minimum of 1.82 individuals km^-2^ and a maximum of 4.32 individuals km^-2^ at 95% confidence level during the study period. The population size was 65 ± 12.6, 61 ± 9.9 and 57 ± 9.2 individuals, with a minimum of 44, 44 and 41 and a maximum of 96, 84 and 79 individuals at the 95% confidence level during 2006–2008 respectively (Table 1). The maximum number of sightings was recorded between 0600 and 0630 hours at distances between 100 m and 200 m (Fig 1A and Fig 1B). Results of the regression analysis for the period of 2003–08 (*p* < 0.05, R^2^ = 0.939) showed a 17% decline in population (Fig 2A). For the period 2006–08 (*p* < 0.05, R^2^ = 0.992) the decline was 8% annum^-^^1^ (Fig 2B). The analysis of the combined period 2003–2018 showed a significant increase in the population (*p*<0.05, R^2^ = 0.608) at the rate of 10% annum^-^^1^.

**Table 1:** Density estimate of hog deer (2006–08) in Keibul Lamjao National Park, India.

Parameters	Estimates			% CV			95% CI					
							Minimum			Maximum		
	2006	2007	2008	2006	2007	2008	2006	2007	2008	2006	2007	2008
p*	0.38 ± 0.58	0.31 ± 0.29	0.30 ± 0.25	15.6	9.59	8.48	0.27	0.25	0.25	0.52	0.37	0.38
n/k*	0.36 ± 0.33	0.22 ± 0.20	0.24 ± 0.20	9.33	10.3	10.5	0.29	0.18	0.18	0.44	0.27	0.28
EDR*	245 ± 19.2	240 ± 11.5	241 ± 10.2	7.83	4.8	4.24	208.9	217.8	220.8	287.8	265.6	263
DS*	1.92 ±0.35	1.19 ± 0.17	1.25 ± 0.17	18.2	14.1	13.5	1.33	0.90	0.95	2.76	1.59	1.63
ES*	1.53 ± 0.10	2.29 ± 0.18	2.02 ± 0.18	6.74	8.2	8.87	1.33	1.93	1.68	1.75	2.72	2.42
D*	2.94 ± 0.57	2.75 ± 0.44	2.51 ± 0.40	19.46	16.3	16.2	1.99	1.99	1.82	4.32	3.80	3.46
N*	65 ± 12.6	61 ± 9.9	57 ± 9.2	19.46	16.3	16.2	44	44	41	96	84	79

*p, probability of detection under the curve; n/k, encounter rate; EDR, effective detection radius; DS, group density; ES, group size; D, individual density; N, population.

**Fig 3:**
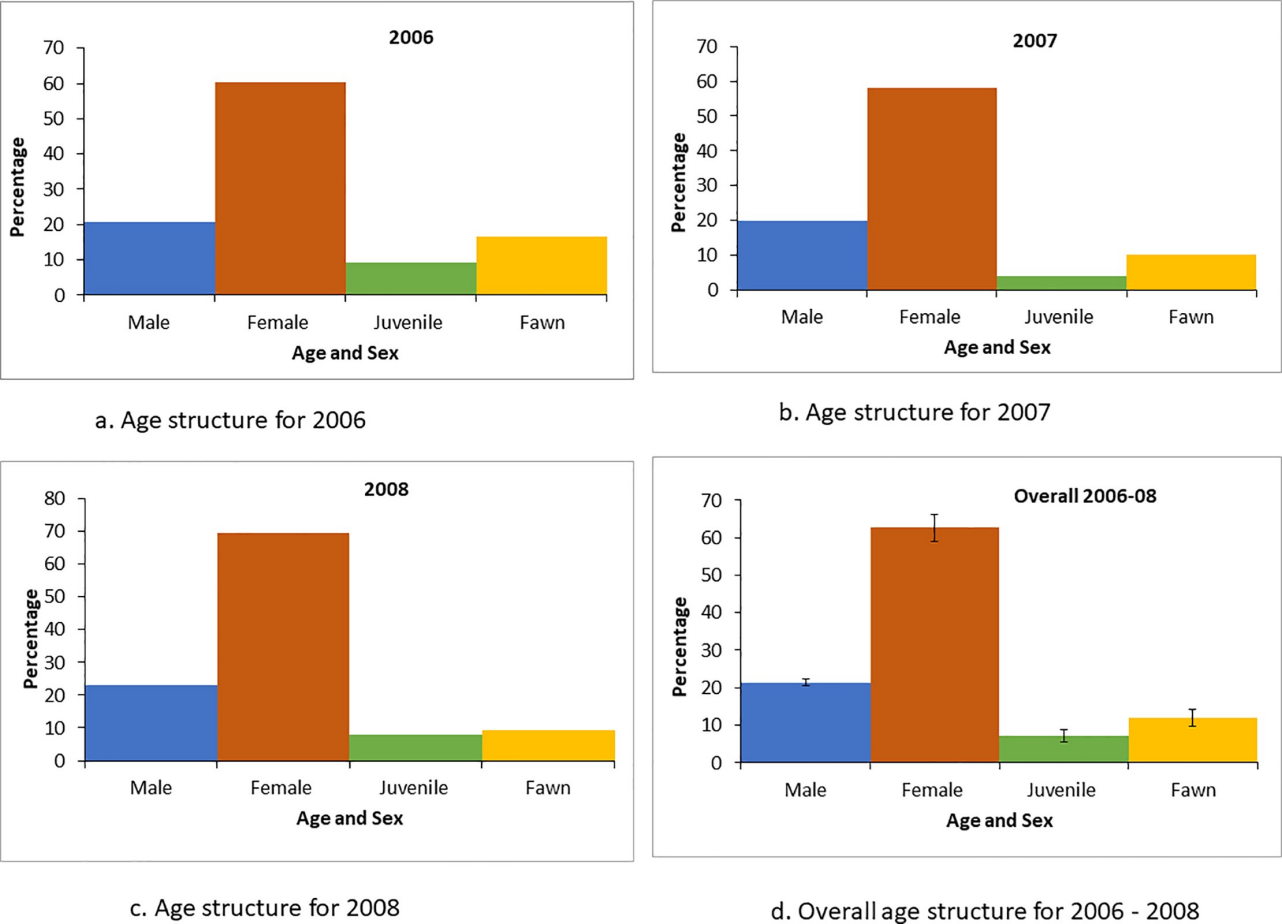
Age structure of hog deer population during population estimation in Keibul Lamjao National Park, India. (a) Age structure for 2006, (b) Age structure for 2007, (c) Age structure for 2008 and (d) Overall age structure for 2006–2008.

The population structure was skewed towards adult females. The number of females was higher as compared to males, juveniles and fawns (Fig 3A and 3D). The overall numbers of adult males, adult females and juveniles fluctuated, whereas the number of fawns declined steadily (Fig 3A–3C). The adult sex ratios were 34.2, 34.5 and 39.9 males per 100 females and the doe-to-fawn ratios were 16.4, 17.2 and 15.8 fawns per 100 females respectively, during the study period. Overall, for the three study years, the male-to-female ratio was 36.2 ± 1.9 males per 100 females, and the doe-to-fawn ratio was 16.5 ± 0.4 fawns per 100 females respectively (Table 2).

**Table 2:** Estimated numbers of hog deer in the various age and sex classes on the basis of the relative proportions seen during the population estimation exercise conducted in 2006–08 in Keibul Lamjao National Park, India and the estimated total population size.

Year	Adult male			Adult female			Juvenile			Fawn		
	Mean	95% lower CI	95% upper CI	Mean	95% lower CI	95% upper CI	Mean	95% lower CI	95% upper CI	Mean	95% lower CI	95% upper CI
2006	13	9	20	39	27	58	6	4	9	11	7	16
2007	12	9	17	35	26	49	2	2	3	6	4	8
2008	13	9	18	40	28	55	5	3	6	5	4	7
Mean	13	9	18	38	27	54	4	3	6	7	5	11
SEM	0.37	0.19	0.88	1.34	0.86	2.69	1.01	0.66	1.55	1.67	1.05	2.64

### Genetic diversity in the wild

Twenty-seven unique individuals were identified from the 38 biological samples collected, and used for further analysis (Table 3). Of the 25 microsatellites screened, 23 were polymorphic, and two loci were non-amplified. The genetic variability study of the hog deer population (n = 27) showed that the number of alleles observed across the 23 loci varied from 2 to 5, whereas the mean number of alleles per locus was 2.70 ± 0.18. The average observed heterozygosity (Ho) was estimated at 0.42 ± 0.02, and the expected heterozygosity (He) was estimated at 0.51 ± 0.03. The mean PIC value was 0.43 ± 0.03 (Table 3). Out of the 23 microsatellites, no deviation from the HWE was observed (Table 3).

**Table 3:** Details of microsatellite loci tested on wild population of *Axis porcinus* at Keibul Lamjao National Park, India (a total of 27 individual samples were used in this study).

Name of locus	Repeat type	Size range	T_a_^†^	N_A_^†^	Ho/He^†^	HWE/LD^†^	PIC^†^	PID _ID (Sibs)_†	Fixation Index (F)	Reference
Ca18*	Di	194–212	52	5	0.63/0.73	Yes/No	0.675	0.421	0.123	Gaur et al. 2003 [43]
T507*	Tetra	174–186	58	3	0.44/0.65	Yes/No	0.568	0.479	0.308	Jones et al. 2002 [44]
ILSTS005*	Di	180–182	50	2	0.577/0.49	Yes/No	0.366	0.605	-0.198	Zhang et al. 2005 [45]
BM4208*	Di	162–164	54	2	0.370/0.475	Yes/No	0.358	0.615	0.206	Say et al 2005 [46]
T123*	Tetra	148–152	58	2	0.259/0.23	Yes/No	0.2	0.793	-0.149	Jones et al. 2002 [44]
T108*	Tetra	128–154	58	2	0.556/0.51	Yes/No	0.375	0.593	-0.111	Jones et al. 2002 [44]
Ca42*	Di	164–178	60	2	0.407/0.44	Yes/No	0.338	0.638	0.056	Gaur et al. 2003 [43]
OarFCB193*	Di	100–126	54	2	0.26/0.33	Yes/No	0.272	0.715	0.201	DeYoung et al 2003 [47]
Cervid1*	Di	144–168	54	4	0.56/0.71	Yes/No	0.642	0.437	0.204	DeYoung et al 2003 [47]
BM6506*	Di	198–200	54	2	0.37/0.45	Yes/No	0.346	0.629	0.167	Anderson et al 2002 [48]
RT27*	Di	130–144	56	2	0.33/0.41	Yes/No	0.321	0.659	0.169	Poetsch et al. 2001 [49]
CelJP27	Di	154–164	59	4	0.519/0.72	Yes/No	0.647	0.434	0.264	Coulson et al 1998 [50]
MAF70	Di	124–128	50	3	0.44/0.64	Yes/No	0.559	0.486	0.296	Zhang et al. 2005 [45]
RT6	Di	98–104	50	3	0.407/0.6	Yes/No	0.526	0.512	0.312	Poetsch et al. 2001 [49]
BM4107	Di	152–190	50	3	0.33/0.51	Yes/No	0.449	0.575	0.333	Zhang et al. 2005 [45]
T193	Tetra	176–186	59	3	0.407/0.53	Yes/No	0.443	0.566	0.219	Jones et al. 2002 [44]
RT1	Di	206–208	54	2	0.26/0.23	Yes/No	0.2	0.793	-0.149	Poetsch et al. 2001 [49]
T156	Tetra	128–130	59	2	0.35/0.45	Yes/No	0.343	0.632	0.213	Jones et al. 2002 [44]
CSSM16	Di	164–166	55	2	0.407/0.33	Yes/No	0.272	0.715	-0.256	Zhang et al. 2005 [45]
INRA011	Di	212–220	54	3	0.444/0.56	Yes/No	0.449	0.55	0.193	DeYoung et al 2003 [47]
D-F/R	Di	154–190	54	3	0.5/0.68	Yes/No	0.588	0.466	0.245	Anderson et al 2002 [48]
INRABERN185	Di	102–114	52	4	0.41/0.66	Yes/No	0.675	0.473	0.373	Zhang et al. 2005 [45]
NVHRT48	Di	102–106	52	2	0.37/0.43	Yes/No	0.33	0.648	0.112	Poetsch et al. 2001 [49]
				**2.70** **± 0.18**	**0.42 ±0.02** **/0.51 ± 0.03**		**0.43 ± 0.03**	**0.58 ± 0.02**	**0.136** **±0.038**	

*Loci required for unambiguous individual identification.

^†^Ho, observed heterozygosity; He, expected heterozygosity; PIC, polymorphic information content; HWE, Hardy-Weinberg equilibrium; PID, probability of identity, the probability that two different individuals will share the same multilocus genotype at a given number of loci; PID_ID(Sibs)_, the probability that a pair of siblings will share the same genotype; Ta, annealing temperature; N_A_, number of alleles; LD, linkage disequilibrium.

### Bottleneck detection

Of the 23 loci found to be polymorphic in the wild population, only seven loci showed a significant heterozygosity excess “S3 Table”. Overall, the Wilcoxon test showed no evidence of a bottleneck in the past (*p =* 0.894 for IAM; *p =* 0.993 for TPM; *p =* 0.999 for SMM). The global analysis indicated no significant heterozygosity excess (He < Heq): 0.252 ± 0.025 < 0.284 ± 0.018 (under IAM); 0.252 ± 0.025 < 0.323 ± 0.021 (under TPM); 0.252 ± 0.025 < 0.359 ± 0.026 (under SMM). The hog deer population did not encounter any genetic bottleneck in the recent past as the allele frequency distribution had an approximately L-shaped curve (as expected under a mutation–drift equilibrium). Although this population is small and isolated, and has gradually declined in recent years, the selected microsatellite loci indicates that it has retained moderate level of heterozygosity.

## Discussion

KLNP lies in the Indo-Burma biodiversity hotspot, one of the most biologically rich regions of the world, with only 5% of the natural habitat of the hotspot remaining [51, 52]. The Indo-Burma hotspot is also one of the most densely populated regions. Thus, it has experienced rapid economic development and changing consumption patterns, which has put the natural ecosystems of the hotspot under immense anthropogenic pressure. KLNP is a small area within this hotspot that is under tremendous anthropogenic pressure. The hog deer population in the park is facing conservation issues such as poaching, predation and competition with domestic livestock for grazing. Furthermore, the hog deer population is threatened by the periodic shrinkage of habitat due to floods, habitat degradation and a lack of connectivity with other viable habitats in the landscape.

The hog deer is typically shy, nocturnal in habit [53] and highly sensitive to human disturbances [54]. The declines in its population are attributed to large-scale transformations in the native range, mainly due to agricultural developments [1]. Considering the increasing anthropogenic pressures in KLNP, the long-term viability of this small population of hog deer seems uncertain. Moreover, the extent of only 40 km^2^ of KLNP dominated by floating meadows and open waters [6, 19], appears to be insufficient to support population expansion. This necessitates conservation and management efforts towards securing a viable population of hog deer in KLNP.

The overall population structure indicated a higher number of females compared with males, juveniles and fawns, which may contribute in limiting the population size. High proportion of females in the population can also be attributed to several natural and anthropogenic factors. With the declining thickness of *phumdis*, the floating vegetation can barely support the weight of adult male hog deer, subsequently affecting the survival rate of male individuals. The antlers of the males occasionally get entangled in the thick vegetation that dominates the habitat, resulting in high male mortality. In addition, traditional hunting practiced by the local communities using snares occasionally entraps the antlers, contributing to increased male mortality. Targeted poaching of male individuals for the aesthetic value of their antlers and trophies may also be a reason for the low male counts. Instances of male mortality due to these factors were recorded at times during the study period. The reduced numbers of fawns to female ratio in the park can be attributed to predation by dogs and wild boar, which has been observed during the course of the study and also from other reports (e.g. [55]). The present study suggest an initial declining trend in the population in the park and subsequent increase from 2013–2018, which may be a consequence of active conservation measures.

The consequences of habitat fragmentation on the genetic structure and variability are still not well-studied [56]. However, several studies have pointed out that fragmented and isolated population such as the hog deer in KLNP may be susceptible to the increase in population differentiation caused by loss of heterozygosity, genetic drift and inbreeding depression [9, 10, 57, 58]. Long term survival of a species with small and declining population is not only limited by genetic diversity but is also vulnerable to natural catastrophes and demographic and environmental stochasticity [59]. With respect to the hog deer population in KLNP, the loss of genetic diversity may not have acute effect on short term population growth, however it may restrict the evolutionary potential of the species by limiting the selection flexibility in the long run [60, 61].

High levels of genetic diversity in a population is also essential to adapt to the changing environment because it increases the probability of individuals in a population to retain variations of alleles which in turn will enable the population to persist for more generations [62]. In our study, the He of the hog deer population was 0.51 ± 0.03, close to the mean He of 0.559 of sika deer in China but much higher than the mean He of the Sichuan sika deer (0.477). It was slightly lower than the mean He of Przewalski’s gazelle (*Procapra przewalskii*) (He = 0.552) [63], Manchurian sika deer (0.584) and South China sika deer (Jiangxi, 0.585; Zhejiang, 0.589) [64]. Compared with other ungulates, such as the red deer (*Cervus elaphus*) (He = 0.78 [65]; He = 0.804 [66]; He = 0.62–0.85 [67]), forest musk deer (*Moschus berezovskii*) (He = 0.8–0.9) [68] and Mongolian wild ass (*Equus hemionus hemionus*) (He = 0.83) [69], the hog deer had a relatively low He value and was equivalent to the milu (*Elaphurus davidianus*) (He = 0.46–0.54) [70]. In our study, the PIC values of eight of the 23 microsatellite loci were higher than 0.5, indicating high polymorphism. The heterozygosity and PIC indicated that the hog deer in KLNP had moderate levels of genetic diversity (Table 3).

Hartl and Clark [71] considered that population deviations from the HWE are mainly due to small populations, non-random mating, gene mutation, migration or other factors. No deviation from the HWE was observed in the selected loci. In addition, due to the relatively closed geographical position of KLNP, *i*.*e*., the lack of connectivity with the surrounding areas, very few hog deer were distributed in its periphery [72]. This indicates that the moderate level of genetic variation in KLNP population compared to other hog deer populations in India [12] may be due to the geographical isolation of the population and lack of connectivity with the adjacent landscape. The gene pool is restricted, and little genetic exchange with other populations is happening due to the unique island-like geography of the area, coupled with dense human habitation around the park. In the present study, the Ho values of selected markers were significantly higher than the He values. When a population experiences a reduction in its effective size, it generally develops a heterozygosity excess at selectively neutral loci [39].

The bottleneck analysis using heterozygosity excess test revealed that the hog deer population of KLNP has not undergone any genetic bottleneck in the recent past. The global analysis indicated no significant heterozygosity excess and therefore, null hypothesis of a mutation–drift equilibrium was rejected, no visible genetic signature of a bottleneck in the recent past. The mode shift test also resulted in a normal L-shaped curve, indicating an absence of genetic bottleneck in hog deer population at KLNP.

This study assessed the population structure and genetic diversity of the small and isolated population of hog deer, and highlighted the need to prioritize conservation measures for the persistence of the population in its natural habitat. Although the population was showing a declining trend, it is showing signs of recovery. This could be attributed to stringent protection measures undertaken by the park authority. However, habitat degradation, poaching and mortalities due to disease still persists in the park. Hence, strict protection measures coupled with scientifically sound management regimes will certainly enhance the long term persistence of the hog deer population in the park.

## Supporting information

S1 Table Total estimated population size of hog deer in Keibul Lamjao National Park, India (2003–2018). (DOCX)

S2 Table Best model analysis of hog deer during population estimation during the study period in Keibul Lamjao National Park, India. (DOCX)

S3 Table Bottleneck detection of hog deer in Keibul Lamjao National Park, India indicating (He < Heq) mutation–drift equilibrium obtained with three mutation models. (DOCX)
