# Supplementary material for: Demographic and genetic structure of a severely fragmented population of the endangered hog deer (Axis porcinus) in the Indo-Burma biodiversity hotspot
Source: PLoS One. 2020 Feb 6;15(2):e0210382. doi: 10.1371/journal.pone.0210382 (PMC7004368; doi:10.1371/journal.pone.0210382)
Supplement: S1 Table — (DOCX) [file pone.0210382.s001.docx]

**SUPPLEMENTARY ONLINE MATERIALS FOR ARTICLE**

**Demographic and genetic structure of a severely fragmented population of the endangered hog deer (*Axis porcinus*) in the Indo-Burma biodiversity hotspot**

Sangeeta Angom, Chongpi Tuboi, Mirza Ghazanfar Ullah Ghazi, Ruchi Badola, Syed Ainul Hussain*

Wildlife Institute of India, Dehra Dun, Uttarakhand, India

*Corresponding author

Email: [hussain@wii.gov.in](mailto:hussain@wii.gov.in)

Tel: +91-9412075660

**Supporting Information File 1**

**Table S1.** Total estimated population size of hog deer in Keibul Lamjao National Park, India (2003-2018)

**Table S1.** Total estimated population size of hog deer in Keibul Lamjao National Park, India (2003-2018)

| **Sl. No** | **Year** | **Method used** | **Estimated population size** | **Source** |
| --- | --- | --- | --- | --- |
| 1. | 2003 | Block Count | 132 | Manipur Forest Department |
| 2. | 2006 | Point Count | 65 | Present Study |
| 3. | 2007 | Point Count | 61 | Present Study |
| 4. | 2008 | Point Count | 57 | Present Study |
| 5. | 2013 | Point Count | 212 | Manipur Forest Department |
| 6. | 2016 | Point Count | 276 | Manipur Forest Department |
| 7. | 2018 | Point Count | 288 | Manipur Forest Department |
