# Supplementary material for: Demographic and genetic structure of a severely fragmented population of the endangered hog deer (Axis porcinus) in the Indo-Burma biodiversity hotspot
Source: PLoS One. 2020 Feb 6;15(2):e0210382. doi: 10.1371/journal.pone.0210382 (PMC7004368; doi:10.1371/journal.pone.0210382)
Supplement: S2 Table — (DOCX) [file pone.0210382.s002.docx]

**SUPPLEMENTARY ONLINE MATERIALS FOR ARTICLE**

**Demographic and genetic structure of a severely fragmented population of the endangered hog deer (*Axis porcinus*) in the Indo-Burma biodiversity hotspot**

Sangeeta Angom, Chongpi Tuboi, Mirza Ghazanfar Ullah Ghazi, Ruchi Badola, Syed Ainul Hussain*

Wildlife Institute of India, Dehra Dun, Uttarakhand, India

*Corresponding author

Email: [hussain@wii.gov.in](mailto:hussain@wii.gov.in)

Tel: +91-9412075660

**Supporting Information File 2**

**Table S2.** Best model analysis of hog deer during population estimation during the study period in Keibul Lamjao National Park, India

**Table S2.** Best model analysis of hog deer during population estimation during the study period in Keibul Lamjao National Park, India

| **Model** | **AIC** | | | **D^†^** | | | **N^†^** | | | ***P*** | | |
| --- | --- | --- | --- | --- | --- | --- | --- | --- | --- | --- | --- | --- |
|  | **2006** | **2007** | **2008** | **2006** | **2007** | **2008** | **2006** | **2007** | **2008** | **2006** | **2007** | **2008** |
| Uniform + simple polynomial | 344 | 305 | 319 | 3.01 | 2.33 | 2.17 | 57 | 47 | 43 | 0.54 | 0.72 | 0.69 |
| Uniform + cosine* | 94 | 72 | 75 | 2.94 | 2.75 | 2.51 | 65 | 61 | 57 | 0.91 | 0.97 | 0.95 |
| Half normal + cosine | 345 | 301 | 313 | 2.87 | 2.41 | 2.53 | 54 | 49 | 51 | 0.34 | 0.44 | 0.21 |
| Half normal + simple polynomial | 346 | 301 | 313 | 2.88 | 2.42 | 2.54 | 54 | 49 | 51 | 0.53 | 0.38 | 0.66 |
| Half- normal + Hermite polynomial | 345 | 301 | 313 | 2.87 | 2.41 | 2.53 | 54 | 49 | 51 | 0.57 | 0.70 | 0.48 |
| Hazard rate + cosine | 348 | 302 | 313 | 2.50 | 2.83 | 2.65 | 47 | 57 | 53 | 0.49 | 0.55 | 0.58 |
| Hazard rate + Hermite polynomial | 346 | 302 | 313 | 2.50 | 2.83 | 2.65 | 47 | 57 | 53 | 0.61 | 0.58 | 0.38 |
| Hazard rate + simple polynomial | 346 | 302 | 313 | 2.50 | 2.94 | 2.65 | 47 | 56 | 53 | 0.68 | 0.33 | 0.43 |

*Uniform + cosine is the best fitted model on the basis of AIC value (lowest value). ^†^D, density; N, number.
