# Supplementary material for: Demographic and genetic structure of a severely fragmented population of the endangered hog deer (Axis porcinus) in the Indo-Burma biodiversity hotspot
Source: PLoS One. 2020 Feb 6;15(2):e0210382. doi: 10.1371/journal.pone.0210382 (PMC7004368; doi:10.1371/journal.pone.0210382)
Supplement: S3 Table — (DOCX) [file pone.0210382.s003.docx]

**SUPPLEMENTARY ONLINE MATERIALS FOR ARTICLE**

**Demographic and genetic structure of a severely fragmented population of the endangered hog deer (*Axis porcinus*) in the Indo-Burma biodiversity hotspot**

Sangeeta Angom, Chongpi Tuboi, Mirza Ghazanfar Ullah Ghazi, Ruchi Badola, Syed Ainul Hussain*

Wildlife Institute of India, Dehra Dun, Uttarakhand, India

*Corresponding author

Email: [hussain@wii.gov.in](mailto:hussain@wii.gov.in)

Tel: +91-9412075660

**Supporting Information File 3**

**Table S3.** Bottleneck detection of hog deer in Keibul Lamjao National Park, India indicating (He < Heq) mutation–drift equilibrium obtained with three mutation models

**Table S3.** Bottleneck detection of hog deer in Keibul Lamjao National Park, India indicating (He < Heq) mutation–drift equilibrium obtained with three mutation models

| **Loci** | **Observed** | **IAM*** | | **TPM*** | | **SMM*** | |
| --- | --- | --- | --- | --- | --- | --- | --- |
|  | He* | Heq* | Prob* | Heq* | Prob* | Heq* | Prob* |
| AY302223 | 0.207 | 0.362 | 0.27 | 0.421 | 0.14 | 0.484 | 0.055 |
| BM4208 | 0.331 | 0.211 | 0.298 | 0.24 | 0.358 | 0.265 | 0.402 |
| RT6 | 0.268 | 0.353 | 0.383 | 0.427 | 0.2 | 0.488 | 0.088 |
| INRA011 | 0.234 | 0.368 | 0.295 | 0.406 | 0.182 | 0.488 | 0.063 |
| RT1 | 0.14 | 0.22 | 0.481 | 0.231 | 0.427 | 0.264 | 0.345 |
| Ca42 | 0.171 | 0.215 | 0.511 | 0.238 | 0.461 | 0.243 | 0.45 |
| Cervid1 | 0.326 | 0.48 | 0.212 | 0.542 | 0.085 | 0.606 | 0.024 |
| RT27 | 0.171 | 0.214 | 0.514 | 0.242 | 0.466 | 0.249 | 0.432 |
| NVHRT48 | 0.171 | 0.22 | 0.531 | 0.237 | 0.474 | 0.26 | 0.405 |
| BM6506 | 0.073 | 0.225 | 0.33 | 0.251 | 0.262 | 0.251 | 0.235 |
| OarFCB193 | 0.257 | 0.22 | 0.395 | 0.239 | 0.445 | 0.249 | 0.48 |
| T123 | 0.23 | 0.205 | 0.397 | 0.234 | 0.486 | 0.249 | 0.518 |
| T156 | 0.382 | 0.221 | 0.246 | 0.243 | 0.286 | 0.261 | 0.338 |
| CelJP27 | 0.205 | 0.361 | 0.255 | 0.419 | 0.136 | 0.482 | 0.046 |
| D-F/R | 0.336 | 0.366 | 0.447 | 0.423 | 0.319 | 0.485 | 0.157 |
| T193 | 0.297 | 0.357 | 0.414 | 0.426 | 0.235 | 0.481 | 0.127 |
| T108 | 0.107 | 0.202 | 0.476 | 0.241 | 0.354 | 0.257 | 0.309 |
| T507 | 0.532 | 0.361 | 0.207 | 0.423 | 0.281 | 0.476 | 0.396 |
| BM4107 | 0.208 | 0.372 | 0.275 | 0.424 | 0.136 | 0.485 | 0.047 |
| MAF70 | 0.507 | 0.361 | 0.284 | 0.42 | 0.402 | 0.478 | 0.514 |
| L23481 | 0.292 | 0.211 | 0.335 | 0.235 | 0.402 | 0.262 | 0.468 |
| AF232760 | 0.307 | 0.208 | 0.311 | 0.239 | 0.376 | 0.253 | 0.422 |
| INRABERN185 | 0.037 | 0.212 | 0.232 | 0.235 | 0.175 | 0.252 | 0.17 |
|  | 0.252 ± 0.025 | 0.284 ± 0.018 |  | 0.323 ± 0.021 |  | 0.359 ± 0.026 |  |

*He, expected heterozygosity; Heq, heterozygosity equilibrium; Prob, probability; IAM, infinite allele model; TPM, two-phase model; SMM, stepwise mutation model.
